# Associations between Alternate Healthy Eating Index-2010, Body Composition, Osteoarthritis Severity, and Interleukin-6 in Older Overweight and Obese African American Females with Self-Reported Osteoarthritis

**DOI:** 10.3390/nu11010026

**Published:** 2018-12-22

**Authors:** Macy Mears, Lisa Tussing-Humphreys, Leah Cerwinske, Christy Tangney, Susan L. Hughes, Marian Fitzgibbons, Sandra Gomez-Perez

**Affiliations:** 1 Department of Clinical Nutrition, College of Health Sciences, Rush University, Chicago, IL 60601, USA; mearsmacy@gmail.com (M.M.); Leah_A_Cerwinske@rush.edu (L.C.); Christy_Tangney@rush.edu (C.T.); 2 Institute for Health Research and Policy, University of Illinois at Chicago, Chicago, IL 60601, USA; ltussing@uic.edu (L.T.-H.); shughes@uic.edu (S.L.H.); mlf@uic.edu (M.F.); 3 University of Illinois Cancer Center, University of Illinois at Chicago, Chicago, IL 60601, USA; 4 Department of Medicine, College of Medicine, University of Illinois at Chicago, Chicago, IL 60601, USA; 5 School of Public Health, University of Illinois at Chicago, Chicago, IL 60601, USA

**Keywords:** AHEI-2010, body composition, inflammation, osteoarthritis, obesity, African Americans, older adults

## Abstract

Osteoarthritis (OA) is a leading cause of immobility in the United States and is associated with older age, inflammation, and obesity. Prudent dietary patterns have been associated with disease prevention, yet little evidence exists describing diet quality (DQ) in older overweight or obese African American (AA) adults with OA and its relation to body composition. We conducted a secondary data analysis of a dataset containing alternate Healthy Eating Index-2010 (AHEI-2010), body composition, OA severity, and serum interleukin-6 (IL-6) data from 126 AA females (aged 60–87 years) with OA to examine the relationships between these variables. Our sample had poor DQ and reported having higher OA severity as measured by the Western Ontario and McMaster Universities Osteoarthritis Index (WOMAC). Interleukin-6 was negatively correlated with AHEI-2010, and AHEI-2010 and the WOMAC physical function subcategory (WOMACpf) were significant predictors of IL-6 (odds ratio (OR): 0.95, 95% confidence interval (CI) 0.92–0.99 and 1.04, 95% CI 1.01–1.07, respectively, *p* < 0.05) but not body composition. In conclusion, AHEI-2010 and WOMACpf were significant predictors of inflammation (IL-6) and AHEI-2010 accounted for ~16% of the variation of IL-6 (inflammation) in this sample.

## 1. Introduction

Osteoarthritis (OA), primarily affecting the hip, knee, and hand joints, is the most common type of arthritis with ~40% of adults over the age of 40 having some degree of knee OA [1]. OA is characterized by a breakdown of articular cartilage and low-grade inflammation [2]. It is estimated that 1 in 5 adults in the United States will be 65 years or older by 2030 [3], giving rise to an older population living longer with chronic diseases and ailments such as OA [4]. Maintaining quality of life and health in aging is important and modifiable factors, like diet and exercise, play important roles [5]. Using the National Health and Nutrition Examination Survey (NHANES) III, oversampled for minorities and specific to those 60 years and older, 47.3% of all individuals (50.5% women and 43.1% men) had at least some degree of radiographic evidence of knee OA [6]. Higher radiographic knee OA is reported for African Americans (AA) compared to Mexican-Americans or Non-Hispanic Whites (NHW) (52.4%, 39.7%, and 36.2%, respectively, *p* < 0.01) and the highest prevalence of OA is reported specifically among AA women (60.2%, 95% CI: 52.8–67.5%) [6].

OA is one of the leading causes of immobility in older adults living in the U.S. and is often closely associated and/or exacerbated by obesity [7]. Findings from a meta-analysis of 47 observational studies showed that preventing obesity, often a low-grade inflammatory state [8,9], could reduce the risk of knee OA by up to 50% [10]. Additionally, overweight was associated with two-times higher odds of OA while obesity had almost four times higher odds of OA compared to adults with normal body mass index (BMI) [10]. OA symptoms, including chronic pain, morning stiffness, and sensations of grating joints may also be related to systemic and local (synovial) low-grade inflammation [11,12]. The Western Ontario and McMaster Universities Osteoarthritis Index (WOMAC) score captures the degree of OA symptom severity and has been validated as a self-administered health status measure in adults with hip or knee OA [13]. Similar to weight gain, obesity-driven changes to body fat accumulation and distribution, such as increased proportions of abdominal fat tissue, are also postulated as major contributing factors of low-grade inflammation [14]. Of particular interest, excess visceral adipose tissue (VAT), located in the intra-abdominal region, is consistently associated with inflammation and chronic disease development; however, the exact link between VAT and OA development is unknown [14]. Taken together, the evidence suggests that obesity in some older adults, primarily in the intra-abdominal region, is an important driver of inflammation and OA.

High adherence to dietary patterns such as, the Harvard Healthy Eating Plate, which encourages a diet rich in fruits, vegetables, fish and whole grains, with moderate consumption of alcohol and low intake of sugar-sweetened beverages, is associated with disease prevention and longevity [15]. Quantifying adherence to a given dietary pattern using statistical methods or a scoring index, such as the alternate Healthy Eating Index (AHEI) which assesses adherence to the Harvard Health Eating Plate, allows examination of associations between diet quality (DQ) and health [16].

Little to no evidence exists exploring links between OA, obesity, and dietary patterns and much less in older AA females. Additionally, it is unclear if better DQ has beneficial effects on body composition, such as regional fat distribution defined by VAT or overall body fat percent (BF%) or if it is associated with lower systemic inflammation and lower OA severity, particularly for older AAs with OA. Thus, the purpose of this study was to examine the associations between DQ, measured by the AHEI-2010, body composition, serum interleukin-6 (a biomarker of inflammation), and OA severity in older AA adults with self-reported OA.

## 2. Materials and Methods

### 2.1. Study Design

This study was a cross-sectional analysis of data collected at baseline from a subset of subjects accrued to the Fit & Strong! Plus comparative effectiveness trial, herein referred to as the *parent* study (#R01AG039374) [17,18]. A full description of the *parent* study design and methods have been published elsewhere [17].

An ancillary study to the *parent* study was conducted (American Cancer Society of Illinois, #2617755), to explore changes in circulating biomarkers and body composition from baseline to post-intervention in a subset of subjects [18]. Data collected as part of the *parent* and ancillary studies served as the basis for this analysis to examine relationships between DQ (AHEI-2010), circulating inflammatory marker (IL-6), body fat composition, including BF% and VAT, and OA severity in older AA adults with self-reported OA who were overweight or obese.

Briefly, participants were overweight and obese older adults with self-reported OA of the knee or hip, referred to as lower extremity (LE) OA who resided in the Chicagoland area [17]. The ancillary study specifically targeted the AA participants from the first eight iterations of the *parent* trial. Eligibility criteria including: 60 years or older, overweight or obese (BMI 25–50 kg/m^2^), not meeting physical activity guidelines of at least 150 min per week, able to attend interviews and weekly classes, and self-reported LE OA (defined as acknowledging knee or hip region pain most days in the last month or remembering knee, hip, lower back, ankles, or feet pain most days of at least one month in the past six months). Exclusion criteria included: rheumatoid arthritis or other health conditions that contraindicated exercise, uncontrolled diabetes, hip/knee surgery in the past six months, intention for hip/knee surgery within the upcoming year, steroid injections in the previous three months in hip/knee regions, or a score of one or more on an exercise and high-risk screener [17]. Additionally, the ancillary study included individuals who identified as AA, were able to attend University of Illinois at Chicago (UIC) for visits, and were willing to undergo fasting blood tests and dual energy X-ray absorptiometry (DXA) scans. It excluded subjects who had cancer within the past five years, a body weight of >450 pounds (204.5 kg), or those unwilling to refrain from medications that could affect blood biomarker results [18]. All participants provided written informed consent. This secondary analysis using de-identified data was exempt from full Institutional Review Board submission under Policy RA-IRB-118 for non-human subjects research at Rush University.

### 2.2. Demographics, Subject Characteristics, and Anthropometrics

At baseline of the *parent* study, trained research staff collected data in person with a socio-demographic questionnaire (e.g., age, socioeconomic status, education). A medical health history questionnaire to ascertain information about major comorbidities (e.g., diabetes, hypertension, cardiovascular disease) was administered. Height was measured with a portable stadiometer (Seca, Chino, CA) to the nearest 0.5 cm in duplicate following the standard. Body weight was measured using a commercial digital scale (Tanita BWB 800, Arlington Heights, IL, USA) to the nearest 0.1 km in duplicate following standard procedures. Body mass index (BMI) was calculated as weight in kilograms divided by height in meters squared (kg/m^2^). A BMI value 25–29.9 kg/m^2^ was considered overweight and ≥30 kg/m^2^ obese [19].

### 2.3. Severity of Osteoarthritis (OA)

The WOMAC questionnaire, a validated tool in various populations, which is used to measure severity of OA, assigns total scores between 0–96 points based on three separate components of OA severity: physical function (0–68 points), stiffness (0–8 points), and pain (0–20 points) [13]. For this study, total WOMAC scores and the subcomponent scores for physical function (WOMACpf) were analyzed. Higher total WOMAC scores represent worse OA severity, and higher physical function subcomponent score, WOMACpf, (>20) indicate worse physical function. The sample was dichotomized based on highest (scores > 20) versus lowest (scores ≤ 20) WOMACpf subcategory scores (i.e., higher scores are indicative of worse physical function) to explore if there were differences in DQ and/or inflammation between these two severity groups.

### 2.4. Body Composition

Body composition estimates of BF% and VAT were obtained via whole body DXA scan (GE Healthcare iLunar DXA, WI, USA). Subjects were required to fast 8 h prior to the scan, abstain from exercise, and wear light clothing with prohibited items removed from pockets on the day of scan as previously described [17]. A half-body scan, following manufacturer specifications [18], was conducted on participants whose body fell outside of the scan’s field of view.

### 2.5. Circulating Inflammatory Marker

Fasting antecubital venous blood samples were collected by trained phlebotomists and processed for serum following standard methods. IL-6 was assessed using a high sensitivity (hs) enzyme-linked immunosorbent assay (ELISA) (R&D Systems, Minneapolis, MN, USA). Average coefficient of variation for hs-IL-6 was 9.18%.

### 2.6. Diet Quality

Dietary intake was quantified from an interviewer-administered Block 2005 Food Frequency Questionnaires (FFQ) (NutritionQuest, Berkeley, CA, USA) [20,21,22]. The FFQ consisted of 110 questions (food items and supplements). Block 2005 FFQs were sent to NutritionQuest (Berkeley, CA, USA) for analysis including estimates for AHEI-2010. FFQs with total energy intake of <500 and >3500 calories (kcals) per day for females were deemed implausible [23,24]. The original data contained 134 females, but after adjusting for plausible caloric intake, final analysis included 126 females. Details for calculating AHEI-2010 have been published elsewhere [25]. The AHEI-2010 scores can range from 0 to 110 points with higher scores reflecting greater adherence to the Harvard Healthy Eating Plate [25].

### 2.7. Statistical Analysis

The statistical program used was IBM SPSS Statistics for Windows, Version 22 Premium (IBM Corp, Armonk, NY, USA). Continuous variables, including age, total WOMAC score, BMI, BF%, VAT, AHEI-2010, and IL-6, were described using median and interquartile ranges (IQR) or mean ± standard deviation (SD) based on normality. Spearman and partial correlational coefficients were conducted to determine associations between AHEI and body composition (VAT and BF%) and AHEI-2010 and inflammation (IL-6). For variables with significant *p*-values, the magnitude of the association was determined using an effect size chart with the coefficient of determination (*r^2^*) [26]. WOMACpf subcategory, a subcomponent of the overall WOMAC score, was identified as a potential confounder between DQ and inflammation in the sample and included in the models as a covariate. Logistic regression models were conducted to assess the relationship between DQ and inflammation, adjusted for BF% or VAT with high or low outcome variables for IL-6 (median splits based on ≥3.5 pg/mL for hi and <3.5 pg/mL for lo) with low as the reference category. To assess the strength of the relationship between predictors and the prediction, as well as the predictability of logistic regression models, Cox and Snell R squared and Nagelkerke R squared statistics were used. Significance was set at a *p*-value of <0.05.

## 3. Results

### 3.1. Sample Characteristics

A total of 126 AA females were analyzed. Demographic and clinical characteristic data are presented in Table 1. The majority of females attended some or at least four years of college. Average BMI was 35.2 ± 5.8 kg/m^2^. Over 50% of females had BMI ≥ 35 kg/m^2^, indicative of higher severity of obesity in this older adult group. The median BF% was approximately 47% and the median VAT volume was 1494 cm^3^. About 56% of the sample scored higher WOMAC scores indicating higher or worse severity of OA.

Approximately 44% of the sample (Table 1) reported higher WOMACpf scores (i.e., higher scores are indicative of worse physical function). Age, comorbidity distribution, and BF% did not significantly differ between groups. However, those with higher BMI (≥35 kg/m^2^) reported worse physical function. Median VAT volume was greater for those with higher scores (1703 cm^3^ vs. 1309 cm^3^ respectively, *p* = 0.002). IL-6 was significantly higher for women with higher WOMAC scores versus the lower score group, 4.1 pg/mL (0.1, 19.8) vs. 3.3 pg/mL (0.7, 16.3), respectively (*p* = 0.040). There were no significant differences of AHEI-2010 scores between females with high versus low WOMAC scores (55.8 ± 10.2 vs. 58.5 ± 11.2, *p* = 0.171).

### 3.2. Nutrient Intakes and Diet Quality as Measured by Alternate Healthy Eating Index-2010 (AHEI-2010)

Median (interquartile range, IQR) total daily caloric intake of the sample and selected median estimates of dietary nutrients are shown in Table 2. The median overall AHEI-2010 score was 57.4 ± 10.9 points (Table 3). The AHEI-2010 score for median servings of total fruit (not including juice) was low at 3.1 (0.1,10), as well as the score (reverse scored) for sugar-sweetened beverages and fruit juices (1.04 (0,10)), meaning the majority of the females studied consume these beverages daily. No differences in mean AHEI-2010 scores were observed between those females with lower vs. higher WOMAC scores (58.5 ± 11.2 vs. 55.8 ± 10.2, *p* = 0.171).

### 3.3. Associations between Inflammation, Body Composition, OA Severity, and Diet Quality

Results of correlational analysis revealed inverse associations between AHEI-2010 and BF%, as well as for AHEI-2010 and BMI (ρ = −0.20, *p* = 0.03 and ρ = −0.21, *p* = 0.02, respectively). AHEI-2010 and IL-6 were inversely correlated (ρ = −0.23, *p* = 0.009). Positive correlations were found between BMI and IL-6 (ρ = 0.19, *p* = 0.03) as well as BMI and WOMACpf and ρ = 0.22, *p* = 0.02). When adjusting for BF% using partial correlations, there were no significant findings between AHEI-2010 and IL-6.

Results of unconditional logistic regression with IL-6 (high vs. low based on median split of 3.5 pg/mL) as outcome variable revealed a significant inverse association for AHEI-2010 and IL-6 (Table 4). When WOMACpf was included in the model, the inverse association between AHEI-2010 and IL-6 remained (β = −0.04, OR 0.96, CI 0.92–0.99), meaning those with higher DQ had ~4% lower odds of having higher IL-6 based on median split and, as expected, WOMACpf (β = 0.04, OR 1.04, 95% CI 1.00–1.07) was also deemed a significant predictor of IL-6. Higher WOMACpf scores were associated with higher IL-6. VAT and BF% were not significant predictors of inflammation.

## 4. Discussion

Few studies have examined the association between DQ, body composition, OA severity and inflammation in persons with OA, particularly for AA older adults. The main finding of this study was that DQ, measured by AHEI-2010, and OA severity, measured by WOMAC, are significant predictors of systemic inflammation among older AA females with self-reported OA. A similar inverse association between DQ and inflammatory status has been reported by other researchers in various populations but not OA populations [27,28,29,30,31].

### 4.1. Dietary Patterns and Quality of Diet

In our cohort of older overweight and obese AA adults with self-reported OA, overall DQ was less than desirable since reported intakes were lower than nutritional guidelines. This is concerning given inverse associations have been found in the literature between AHEI-2010 score and chronic diseases like cardio-vascular disease (CVD), type 2 diabetes mellitus (T2DM) and chronic inflammation, which are generally more prevalent in AA individuals [6,32,33,34]. Comparing our results to two large prospective studies, researchers of the Nurses’ Health Study (NHS) and the Health Professionals Follow-Up Study (HPFS) found that the highest AHEI-2010 scores (defined by >57.8 points for females in NHS cohort and >62.3 for males in HPFS) were negatively associated with major chronic disease risk, including CVD, T2DM, cancer, and non-traumatic death [25]. Other researchers using data from the National Institute of Health-American Association of Retired Persons (NIH-AARP) study (mean age ~62 years, primarily NHW) found that highest quintile AHEI-2010 scores (60.7–90.7 for females and 60.5–92.1 for males) were associated with lower risk of all-cause, cancer, and CVD mortality among females and males [35]. Another study examining DQ trends of a nationally representative sample (*n* = 29,124, ~20% AA, 52% female, 34% within 60–85 years old), using AHEI-2010 to score DQ, found AA adults had lower AHEI-2010 scores than Mexican-American and NHW groups [36]. However, these differences in DQ did not remain between AA and NHW groups after adjusting for education and income [36]. These findings collectively suggest that DQ is lower for AAs and possibly affected by socioeconomic factors.

### 4.2. Associations between Diet Quality (DQ) and Body Composition

In support of our hypothesis, we found a significant inverse correlation between AHEI-2010 and BF% for females but not between AHEI-2010 and VAT. Using a longitudinal study design, Maskarinec et al. [37] explored the associations of DQ in middle age on BF% and VAT changes in older adulthood among a subgroup of participants from the Multi-ethnic cohort study (60–72 years at recruitment, 17% AA). They found that both male and female participants in the highest tertile of four DQ scores (HEI-2010, AHEI-2010, alternate MED (aMED), and Dietary Approaches to Stop Hypertension (DASH)) at baseline had lower total body fat at follow-up (mean time 20.9 ± 1.2 years) [37]. Participants in the higher tertile of the four DQ indices, including AHEI-2010 at baseline were also less likely to have high VAT at follow-up (HEI-2010 OR: 0.48, 95% CI: 0.35, 0.66; AHEI-2010 OR: 0.62, 95% CI: 0.46, 0.85; aMED OR: 0.65, 95% CI: 0.47, 0.90; DASH OR: 0.41, 95% CI: 0.30, 0.58) [37]. This study provides supporting evidence that DQ can have inverse relationships with both BF% and VAT for both sexes.

### 4.3. Associations between Diet Quality and Inflammation

In support of our stated hypothesis, we found inverse correlations between DQ and inflammation, more specifically AHEI-2010 and IL-6, in overweight and obese females with OA. However, no correlation between DQ and IL-6 existed in females when BF% was adjusted for using partial correlation, suggesting that BF% is an important contributor to inflammation. The correlational observations between AHEI-2010 and IL-6 from our study in AA females align with findings from other studies in various race/ethnic populations. Researchers using a subsample of adult women from the NHS cohort (*n* = 660, 100% female, 43–69 years old) found that of the five DQ indices used (original Healthy Eating Index, original AHEI, Recommended Food Score, Diet Quality Index-Revised, and aMED), AHEI had the strongest inverse associations with inflammatory markers after adjusting for age, BMI, and total kcal intake [27]. Study participants with the highest quintile of AHEI score had 31% lower IL-6 concentrations compared to those in the lowest quintile [27].

### 4.4. Relationships between Diet Quality, Body Composition, Inflammation, and OA Severity

We found notable relationships between DQ, body composition, and OA severity on inflammatory markers in females. Results of our simple logistic regression model suggest that a higher AHEI-2010 score is attributed to a ~4% higher odds (OR 0.953, 95% CI 0.920–0.986, *p* = 0.006) of having “low” IL-6 (<3.5 pg/mL) in females. This adds to the emerging evidence that adherence to prudent dietary patterns may decrease chronic inflammation, and as our study demonstrates, even in populations prone to a higher inflammatory status as in OA. For example, Fung et al. [27] found higher adherence to AHEI-2010 was inversely associated with IL-6 values among females (43–69 years old) in the NHS cohort after adjusting for various demographic variables, physical activity, total kcal intake, and BMI. Overall, existing evidence, including findings from the present study, suggests that higher adherence to various “healthy” dietary patterns can positively impact inflammatory levels among various populations controlling for demographic characteristics (i.e., race, income, total kcal intake), physical activity, and body composition.

Although consistent with emerging evidence, results from our logistic regression model exploring DQ, body composition, and OA severity were modest at best (only predicting ~16%, at most, of the variation in inflammation). This means other factors, known and unknown, which we did not account for could be contributing to inflammation. Collectively, our findings and existing evidence suggest that certain dietary changes to improve DQ may help reduce IL-6 levels in older adults with OA but findings may not be generalizable to all racial groups. Future studies with larger sample sizes are needed to substantiate these findings.

### 4.5. Limitations and Strengths

This study was not without limitations. We used a convenience sample of AA female adults, and our sample size precluded in-depth explorations of the inter-relationships between DQ, body composition, and inflammation. Our study is also limited by the use of only one inflammatory biomarker. We acknowledge that a comparison group consisting of other racial/ethnic groups might have allowed us to better interpret our findings. The cross-sectional design of this study made cause and effect relationships indeterminant. In addition, we scored dietary patterns based on data collected using FFQs which are prone to measurement and reporting errors. Lastly, our findings are only generalizable to urban, overweight and obese, older, AA females with OA.

Despite limitations as listed above, our study had many strengths. Results of our study help fill a gap in the literature related to overweight and obese older AA females with OA and provide descriptive DQ (AHEI-2010) data for this population. In addition, we explored relationships between DQ, body composition, and inflammation which are largely understudied in older AA adults. Data for the study was obtained by trained research personnel using validated measures (i.e., modified Block FFQ, DXA, DQ scores, anthropometric) and standardized protocols.

## 5. Conclusions

In conclusion, AHEI-2010 and WOMACpf were independent predictors of IL-6 suggesting that higher AHEI-2010 scores were related to lower IL-6 taking OA severity into account. Our study contributes to the existing literature by showing that DQ, as defined by AHEI-2010, accounted for ~16% of the variation of IL-6 (inflammation) among older AA adult females with OA. Our findings suggest that higher DQ might help suppress inflammation in older AA females with OA. Further research is needed to explore the additional known and unknown factors (mediators and confounders) contributing to inflammation in AA older adults with OA.

## Figures and Tables

**Table 1:** Demographic and clinical characteristics of sample.

Variables	Females (*n* = 126)
Age, years, median (IQR)	65 (60, 87)
Education Level, *n* (%)	
None or kindergarten	0 (0)
Grades 1–8	1 (0.8)
Grades 9–11	6 (4.8)
Grade 12 or GED	16 (12.7)
College 1–3 years or tech school	54 (42.9)
College 4 years or more	49 (38.9)
WOMAC Total Score, 0–96 points, median (IQR)	24 (1, 86)
WOMACpf Score, *n* (%)	
0–19 points	70 (55.6)
20–68 points	56 (44.4)
Current Health Insurance Coverage ^a^, *n* (%)	
Medicare A	17 (13.5)
Medicare A & B	58 (46)
Medicaid	19 (15.1)
Medicare HMO	17 (13.5)
Private/Supplemental	55 (43.7)
Comorbidities ^b^, yes, *n* (%)	
Diabetes	8 (6.3)
Stroke/Paralysis	6 (4.8)
Heart Disease	2 (1.6)
High Blood Pressure	30 (23.8)
High Cholesterol	18 (14.3)
Cancer	5 (4)
Body Mass Index, kg/m^2^, mean ± SD	35.2 ± 5.8
BMI Category, *n* (%)	
<35 kg/m^2^	62 (49.2)
≥35 kg/m^2^	64 (50.8)
Body Fat Percentage, median (IQR)	46.7 (30.2, 57.2)
Visceral Adipose Tissue volume, (cm^3^), median (IQR)	1494 (53, 3504)
Interleukin-6, pg/mL, median (IQR)	3.5 (0.1, 19.8)

IQR: interquartile range, GED: general education development, WOMAC: Western Ontario and McMaster Universities Osteoarthritis Index, used to determine severity of osteoarthritis (OA) symptoms (96 points = worst severity), WOMACpf: Western Ontario and McMaster Universities Osteoarthritis Index physical function category (20–68 points = worse physical function), HMO: health maintenance organization, BMI: body mass index. ^a^ Missing data for current health insurance coverage for 5 females (4%); ^b^ Missing data for comorbidities for 91 females (72.2%).

**Table 2:** Nutrient intake data of females.

Variables	Females (*n* = 126)
Total Daily Calories, kcals	1389 (892)
% daily kcals from carbohydrates	46.9 ± 8.8
% daily kcals from protein	14.9 (4)
% daily kcals from fat	39.5 (7)
% daily kcals from sweets	15.4 (15)
% daily kcals from alcohol	0 (1.05)
Total MUFA, %	15.6 (5.3)
Total PUFA, %	9.5 (3.1)
Total SFA, %	11.0 (2.8)
Total Trans, %	1.1 (0.4)
Sodium, mg	2156.3 (1521)

Kcals = calories, g=grams, MUFA = monounsaturated fatty acids, PUFA = polyunsaturated fatty acids, SFA = saturated fatty acids, Trans = trans fatty acids.

**Table 3:** Alternate Healthy Eating Index-2010 total and component scores.

	Intake for Max Score	Point Range (max)	Sample Median (IQR) (*n* = 126)
Whole Grains	Women ≥ 75 g	0–10 (10)	1.7 (0.1, 8.5)
Total Fruit (not juice)	≥4 servings	0–10 (10)	3.1 (0.1, 10)
SSB and Fruit Juices	0 servings	0–10 (10)	1.04 (0, 10)
Vegetables (excluding potatoes)	≥5 servings	0–10 (10)	5.11 (0.3, 10)
Nuts and Legumes	≥1 serving	0–10 (10)	3.7 (0.2, 10)
Red and Processed Meat	0 servings	0–10 (10)	7.4 (0, 10)
EPA + DHA	≥250 milligrams	0–10 (10)	3.0 (0.03, 10)
PUFA	≥10% of total energy intake	0–10 (10)	9.3 (2.6, 10)
Sodium	Lowest decile of sample population	0–10 (10)	7.8 (0, 10)
Alcohol (drinks/day)	Women (0.5–1.5)	0–10 (10)	5 (0, 10)
Trans Fat	≤0.5% of total energy intake	0–10 (10)	8.3 (3.1, 10)
Total AHEI score (0–110 points), mean ± SD			57.4 ± 10.9

IQR= interquartile range, AHEI = Alternate Healthy Eating Index-2010; max = maximum, g = grams, SSB = sugar sweetened beverages, PUFA = polyunsaturated fatty acids, EPA = eicosapentaenoic acid, DHA = docosahexaenoic acid, SD = standard deviation.

**Table 4:** Impact of diet quality (AHEI-2010), body composition, and osteoarthritis severity (WOMACpf) on high interleukin-6 concentrations in females (*n* = 126).

Variables	Model 1	Model 2	Model 3	Model 4	Model 5
AHEI-2010	0.95 (0.92–0.99) *	0.96 (0.92–0.99) *	0.96 (0.92–0.99) *	0.95 (0.92–0.99) *	0.96 (0.92–0.99) *
VAT	--	1.00 (1.0–1.0)	1.00 (1.0–1.0)	--	--
BF%	--	--	--	1.00 (0.93–1.07)	0.96 (0.93–1.07)
WOMACpf	--	--	1.04 (1.01–1.07) *	--	1.04 (1.01–1.07) *

Data represented as odds ratio with (95% confidence intervals). * Significant *p*-value set at <0.05. IL-6 = interleukin 6 (median split at ≥3.5 pg/mL for high IL-6 and <3.5 pg/mL for low IL-6 as outcome variable), VAT = visceral adipose tissue volume, AHEI-2010 = Alternate Healthy Eating Index-2010, WOMACpf = Western Ontario and McMaster Universities Osteoarthritis Index (osteoarthritis severity) physical function subcategory, BF% = body fat percentage.
